# Drosophila Protein Kinase CK2: Genetics, Regulatory Complexity and Emerging Roles during Development

**DOI:** 10.3390/ph10010004

**Published:** 2016-12-29

**Authors:** Mohna Bandyopadhyay, Scott Arbet, Clifton P. Bishop, Ashok P. Bidwai

**Affiliations:** Department of Biology, West Virginia University, Morgantown, WV 26506, USA; mohnab04@gmail.com or bandyopadhyaym@hollins.edu (M.B.); sarbet@mix.wvu.edu (S.A.); cbishop@wvu.edu (C.P.B.)

**Keywords:** CK2, Drosophila, Notch, eye development, neurogenesis

## Abstract

CK2 is a Ser/Thr protein kinase that is highly conserved amongst all eukaryotes. It is a well-known oncogenic kinase that regulates vital cell autonomous functions and animal development. Genetic studies in the fruit fly Drosophila are providing unique insights into the roles of CK2 in cell signaling, embryogenesis, organogenesis, neurogenesis, and the circadian clock, and are revealing hitherto unknown complexities in CK2 functions and regulation. Here, we review Drosophila CK2 with respect to its structure, subunit diversity, potential mechanisms of regulation, developmental abnormalities linked to mutations in the gene encoding CK2 subunits, and emerging roles in multiple aspects of eye development. We examine the Drosophila CK2 “interaction map” and the eye-specific “transcriptome” databases, which raise the prospect that this protein kinase has many additional targets in the developing eye. We discuss the possibility that CK2 functions during early retinal neurogenesis in Drosophila and mammals bear greater similarity than has been recognized, and that this conservation may extend to other developmental programs. Together, these studies underscore the immense power of the Drosophila model organism to provide new insights and avenues to further investigate developmentally relevant targets of this protein kinase.

## 1. General Overview

Protein phosphorylation is recognized to be a fundamental and evolutionarily conserved regulatory mechanism that controls virtually all aspects of cell and developmental biology. Despite knowledge of the existence of phospho-proteins, the nature of the participatory enzymes remained unknown until the early 1950s, when the laboratory of Eugene Kennedy described, for the first time, the presence of an enzyme, which they called a protein “phosphokinase” [1]. This enzyme possessed the capacity to transfer a phosphate group from ATP to proteins and resulted in the formation of phospho-serine, known to be highly enriched in casein. In addition, they demonstrated that this enzyme preferentially phosphorylated α-casein, as compared to β-casein. In a sense, these seminal studies of Burnett and Kennedy not only revealed the enzymatic basis for the covalent attachment of phosphate to proteins, but also raised the possibility that this type of enzyme exhibited substrate-specificity, now acknowledged to be a fundamental and defining feature of all members of the protein kinase family. Despite the profound implications of their findings, further studies on this “phosphokinase” were not pursued. Just four years later, Edwin Krebs and Edmond Fischer reported the seminal and landmark discovery that phosphorylation controls enzymatic activity [2], subsequently recognized by the Nobel Prize in 1992 [3]. Recalling the decision to not pursue further studies on “phosphokinases”, and in a perspectives article in 1992, Eugene Kennedy stated that “*I dropped the study of protein kinases, and like the base Indian, cast a pearl away, else richer than all his tribe*” [4]. The identity of the protein kinase(s) described by Burnett and Kennedy remained unknown. Subsequent purification and identification of the participatory enzymes resulted in their naming as “casein kinase(s)”, a misnomer because these enzymes do not reside in the Golgi apparatus, a prerequisite for phosphorylation of the secreted protein casein. These aspects will not be discussed here, given the detailed and excellent historical perspectives (for reviews on CK2, see References [5,6,7,8,9]). The casein-modifying activity has now been definitively linked to the Fam20C protein kinase, that not only phosphorylates casein but is also responsible for the generation of most of the secreted phospho-proteome [10,11]. To remove confusion, the two enzymes Casein Kinase I and II were renamed protein kinase CK1 and CK2. We use this more recent nomenclature, but note that many reports and genome/proteome databases still use the old name.

This review focuses on protein kinase CK2 from the fruit fly *Drosophila melanogaster*, a preeminent animal model, which has illuminated many fundamental principles underlying cell signaling, regulation of gene expression and animal development. We review the subunit composition of Drosophila CK2, the complexity of its gene structure, the multiplicity of its physiological targets that are supported by genetic analyses, physiological/developmental processes revealed by analysis of CK2 mutant flies, large scale screens that have identified proteins that interact with individual CK2 subunits, and conclude with its emerging roles in multiple aspects of eye development.

## 2. Biochemical Properties and Regulation of Dm-CK2

Drosophila CK2 (henceforth abbreviated as Dm-CK2) was first purified to homogeneity by Glover and co-workers, who demonstrated that the enzyme purified from 0 to 18 h old embryos, like its mammalian counterpart [12,13,14], is composed of two catalytic Dm-CK2-α and two regulatory Dm-CK2-β subunits that form a hetero-tetrameric (α2β2) holoenzyme. This enzyme utilizes ATP or GTP with almost equal efficiency, appears to be messenger-independent, auto-phosphorylates Dm-CK2-β, modifies Ser or Thr residues in its targets, and is inhibited by Heparin [15,16]. In addition, Dm-CK2 modifies hyper-acidic regions in target proteins, a property that was first described for the mammalian enzyme from various sources [17,18,19,20]. However, unlike mammalian CK2, which contains two distinct catalytic subunits (α and α′ [21,22]), Dm-CK2 contains a single α isoform. In contrast, metazoan organisms generally contain a single isoform of CK2-β, which was thought to also be the case with Dm-CK2 purified from embryos [15]. However, more recent studies are revealing that the Drosophila genome encodes for the greatest diversity of CK2-β subunit isoforms (see below). These features are also associated with CK2 purified from the yeast *Saccharomyces cerevisiae* [14,16,23,24,25,26,27,28,29]. The first evidence that CK2 is conserved through evolution came from the findings that antibodies raised against mammalian (bovine) CK2 strongly cross-reacted with the corresponding subunits of Dm-CK2 [30]. The cloning of cDNAs encoding Dm-CK2-α and CK2-β subunits by the laboratory of Claiborne Glover [31] revealed the primary sequences of these two subunits, thereby enabling the characterization of the cDNAs of the corresponding subunits from diverse species including budding yeast [32,33], fission yeast [34], nematodes [35,36,37], plants [38], amphibians [39], and mammals [40,41,42,43]. Together, these studies not only reinforced the high conservation of CK2 throughout eukaryotic evolution, but revealed subunit heterogeneity that is unique to each taxonomic group.

Despite a search for regulation through second messengers, small ligands, or phosphorylation, the mechanisms influencing CK2 activity have remained unknown. Consequently, CK2 is generally regarded as a second-messenger independent and constitutively active protein kinase. Regulation through holoenzyme (α2β2 tetramer) dissociation seems unlikely, because monomeric Dm-CK2-α (generated by biochemical or recombinant approaches) displays approximately 25% of the activity seen with the holoenzyme [44,45,46], and addition of CK2-β stimulates activity four-fold concomitant with reconstitution of the tetrameric holoenzyme. Similar findings have been reported for monomeric CK2-α subunits from other sources [27,47]. Regulation through polyamines has been well described [48,49,50,51,52]; these compounds affect Dm-CK2 activity against specific target proteins in vitro, generally act through interactions with the CK2-β subunits [44], and have now been proposed to link CK2 activity to the EGFR/MAPK signaling pathway [53]. However, the broader implications of these findings and their physiological relevance remain unresolved. The findings that CK2 levels and activity respond to mitogen signaling, e.g., Epidermal Growth Factor [54,55], led to broad interest because CK2 functions could be linked to EGFR signaling, a pathway known to be intricately linked to cancer and animal development [56,57,58]. However, a detailed reevaluation reveals that these earlier findings on induction by mitogens were artefactual [59]. Further interest stemmed from the findings that CK2 levels/activity correlate to leukemic transformations [60,61], and other cancers, but whether this involves aberrant CK2 regulation remains unknown.

A novel regulatory mechanism has been recently proposed, and extends an observation, first reported by Claiborne Glover, that Dm-CK2 undergoes polymerization involving an ordered but reversible association of the tetrameric holoenzyme into a filamentous state [62]. Filament formation is favored at physiological ionic strengths, and the filaments dissociate into the tetrameric holoenzyme at ionic strengths that are optimal for CK2 activity in vitro. This property is not seen with the monomeric Dm-CK2-α subunit either expressed in bacteria, yeast or insect cell culture (Bidwai, unpublished), suggesting that tetramer–tetramer associations involve the regulatory CK2β subunit. Indeed, it has recently been demonstrated that human CK2 also undergoes polymerization [63] in a CK2β subunit-dependent manner, raising the prospect that the earlier findings on Dm-CK2 may have broader impact. CK2 filaments do not appear to involve structural rearrangements akin to a “prion-type” polymerization, and the filamentous state is proposed to down-regulate CK2 activity. However, the biological relevance of the CK2 filaments in their native (in vivo) milieu remains to be investigated. A robust in vivo evaluation of this property is now possible given the availability of null and hypomorphic mutants for the *Dm-CK2-α/β* genes (see below) and the identification of interfacial residues that mediate filament formation.

## 3. Substrates of Dm-CK2

The first substrate for Dm-CK2 to be identified was DNA Topoisomerase II [64,65], whose activity was stimulated three-fold upon phosphorylation. This post translational modification (PTM) occurs in vitro and in vivo (Drosophila embryonic Kc cells in culture), findings that have been corroborated for DNA Topoisomerase II from the yeast *S. cerevisiae* [66,67,68]. The application of high throughput whole cell proteomic strategies has enabled detailed analysis of the CK2-dependent cellular phospho-proteome. This approach has successfully been applied to mammalian cells or yeast expressing heterologous proteins upon treatment with high-affinity and -specificity inhibitors of CK2 [69,70], and reveals a multitude of proteins whose phosphorylation is stimulated as well as inhibited, and includes many proteins with cell-autonomous roles. While such information can illuminate the extent of the CK2 phospho-proteome, analyses of cell lines do not reveal targeting of developmentally important genes, whose expression is often restricted to a specific cell-type, a region of the developing embryo/animal, or is under temporal control. Accordingly, Giot and coworkers used the yeast two hybrid strategy to evaluate Drosophila protein–protein interactions at a global level [71], but these interactions have not been subject to follow-up studies. These latter two aspects have restricted our understanding of the CK2 “phospho-proteome” relevant to animal (Drosophila) development, and much of our knowledge of specific targets has emerged on a case-by-case basis (see below).

Compared to mammalian cell-based strategies, studies in Drosophila have identified a smaller cohort of CK2 substrates, and many of these have revealed important roles in development. We used the Drosophila database (Flybase, release date of 18 October 2016), and identified proteins whose interaction with CK2 was revealed by studies that combined direct biochemical with reverse-genetic approaches. We note that the role of Dm-CK2 has also been inferred based on genetic screens using RNAi against Dm-CK2-α/β subunits or via dominant-negative (DN) constructs against Dm-CK2-α, and these studies implicate roles in cell signaling, development, tissue morphogenesis and organogenesis. However, we have excluded these from consideration in this review, because molecular target(s) cannot be discerned solely by “reverse-genetic” approaches. We correlated known targets to the Drosophila-Protein-Interaction-Map (DPIM) database [72,73], an LC-MS based approach in which Dm-CK2 interacting proteins were identified following its expression and co-immunoprecipitation from Drosophila S2 cells (see below). It should, however, be noted that follow-up studies to identify which of these proteins is an interacting partner and/or a substrate for phosphorylation are generally lacking, and therefore only those proteins for which direct biochemical evidence has been reported are included in Table 1.

As shown in Table 1, this list of bona fide Dm-CK2 target proteins is not as extensive as that in mammals. Nevertheless, this list includes numerous developmentally important transcription factors, proteins involved in regulation of cytoskeletal and chromatin structure, ribosome structure and biogenesis, DNA-replication, circadian rhythms, etc. With few exceptions (such as CREB2 [74], Raf [53], Topo-II [64], and rPL22 [75]), most of the proteins in this list serve well-established roles in development. Examples are, Ankyrin-2 [76], Antennapedia [77], Armadillo [78], Cactus [79], Clock [80], Dishevelled [81], dMI-2 [82], E(spl)-M5/M7/M8 [83,84], Engrailed [85], Enhancer of Rudimentary [86], FMR1 [87], GAGA factor-519 [88], Groucho [89], Hairy [90], Heterochromatin protein HP1 [91], mushroom body miniature [92], NAP1 [93], Odd [94], Orb [95], P element Somatic Inhibitor [96], Ribosomal S6 kinase [97], Smoothened [98], Syntaxin-1 [99], and Warts [100]. Remarkably, only three proteins from this list, dMi-2, FMR1 and DNA-Topo-II, have been also identified in the DPiM database (Table 1 and see below). The low overlap between genetically analyzed proteins targeted by CK2 to those identified in the DPiM database reveals limitations inherent to cell-based assays. Although the S2-cell line is of embryonic origin, it does not fully recapitulate gene expression patterns across a developing embryo/animal. Consequently, many genes whose expression is controlled in a spatially and/or temporally restricted manner are not captured in S2-based assays. For example, although the Notch effector proteins E(spl)-M8/M5/M7 were identified in a yeast two-hybrid screen of a Drosophila (0–18 h) embryo cDNA library [101], and the consequences of CK2-mediated phosphorylation of E(spl)-M8 during neurogenesis (eye and bristle development) are well understood [83], these proteins appear to not be endogenously expressed in S2 cells. This is, perhaps, the greatest weakness of high-throughput proteomic methods to reveal the extent of the CK2 “interactome” that regulates animal development.

## 4. Drosophila Genes Encoding Catalytic (α) and Regulatory (β) Subunits

Unlike most metazoan organisms, Drosophila harbors a single *Dm-CK2-α* gene [31]. On the other hand, and unique to Drosophila, is the presence of multiple genes, both X-linked and autosomal, that encode proteins with high homology with metazoan CK2-β subunits (Table 2), which are functionally non-redundant (see below). These are the X-linked *Dm-CK2-β* [102] and *Stellate* (*Ste*) genes [103,104,105], and the autosomal genes *Dm-CK2-β′* [106] and *Suppressor of Stellate Like* (*SSL*, also called Dm-*CK2-β^Tes^*) [107,108]. The *Stellate* locus is unusual in that it harbors multiple copies of the *Ste* gene; this appears to vary between strains, with perhaps the highest number (~200) in the *D. melanogaster* strain Oregon R. This multi copy *Ste* locus, located in a heterochromatic region of the X-chromosome, is unique in that its expression is testis-specific and normally repressed through the action of a Y-linked *Su(Ste)* gene cluster [109]. Consequently, in X/O males (those lacking the Y chromosome), *Ste* undergoes massive de-repression and the Ste protein accumulates at levels sufficient to form crystalline aggregates that disrupt spermatocyte maturation resulting in loss of fertility. *Ste* gene copy number seems to influence the type of crystals; needle-shaped crystals in strains with low copy number but star-shaped crystals in strains with high copy number. The reasons underlying these differences are unresolved, and it is also unknown if sterility in XO males involves Ste-dependent impairment of endogenous Dm-CK2. In a similar manner, *SSL/Dm-CK2-β^Tes^* was thought to be testis-specific [110]. However, *SSL* transcripts are present in the embryo, although levels markedly increase in a male-specific manner in third-instar-larvae, pupae, and adults [107]. These results demonstrate that *SSL/Dm-CK2-β^Tes^* expression encompasses a greater developmental window, and raise the possibility that this alternative Dm-CK2 subunit may confer distinct functions to CK2 in a sex-specific manner.

In contrast to *Dm-CK2-β*′, *Ste*, and *SSL/CK2-β^Tes^*, all of which encode for a single protein isoform, the *Dm-CK2-β* gene encodes for multiple isoforms due to alternative transcription and splicing. These include five transcripts named CK2-β type-VIIa, -VIIb, -VIIc, -VIId and -VII-VI (Table 2, and see References [102,111]). This diversity of transcript types has so far not been reported for other metazoan organisms. All five transcripts encode the highly conserved core of CK2-β, which includes the well characterized (N-terminal) auto-phosphorylation site and the zinc-finger [112], but differ in the length and sequence heterogeneity of ~15–20 C-terminal residues [111]. Consequently, these variations do not affect interaction with Dm-CK2-α, but significantly impact in vivo activities of the encoded proteins (see below). In a similar vein, *Dm-CK2-β*′, *Ste*, *SSL/CK2-β^Tes^* also interact with Dm-CK2-α and appear competent for forming the tetrameric holoenzyme in vitro, but these alternative *Dm-CK2-β* proteins do not act in a functionally redundant manner with the X-linked *Dm-CK2-β* gene (see below).

## 5. Mutations in Catalytic (α) and Regulatory (β) Subunits

**Figure 1:**
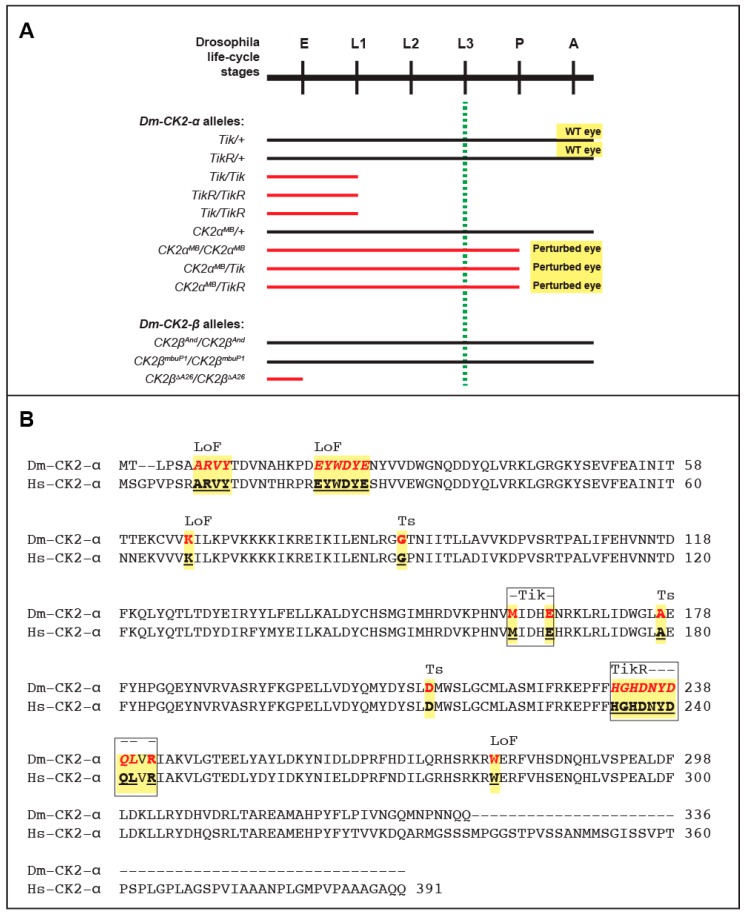
(**A**) Drosophila life cycle stages and effective lethal phase of CK2 mutants. Abbreviations are; E, Embryo; L1, 1st larval stage; L2, 2nd larval stage; L3, 3rd larval stage; P, pupal stage; A, adult. Note that *Dm-CK2-α* mutants *Tik* and *TikR* arrest at the L1 stage, whereas *CK2α^MB^* mutants die at the P-to-A transition. In contrast, *CK2β^And^* and *CK2β^mbuP1^* are viable, whereas *CK2β^∆A26^* die during embryogenesis. Dashed green line denotes stage of life cycle when eye development initiates; black lines denote normal development, whereas red lines denote effective lethal phases of indicated mutant combinations; (**B**) Alignment of CK2-α from *D. melanogaster* (Dm) and *H. sapiens* (Hs). LoF denotes loss-of-function, whereas Ts denotes Temperature-sensitive. The locations of the Tik and TikR mutations are boxed and yellow highlighting denotes conservation of residues between Dm and Hs CK2α subunits.

Mutations in *Dm-CK2-α* were isolated in a genetic screen for dominant modifiers of the circadian clock and resulted in the identification of the first mutant allele called *Timekeeper* (*Tik*). Specifically, *Tik/+* animals display an aberrant circadian clock, whereas *Tik*-homozygotes (*Tik/Tik*) die at the first larval stage ([113], and see Figure 1). A spontaneous and partial revertant of the clock phenotype was also identified; this allele called *TikR* is also lethal at the first larval stage in the homozygous state (Figure 1). The *Tik* allele harbors two missense mutations, M^161^K+E^165^D, and encodes an inactive form of Dm-CK2-α, which retains proper folding because its ability to interact with Dm-CK2-β is indistinguishable from wild-type Dm-CK2-α. In addition, Rasmussen and coworkers have reported that, compared to the wild type enzyme, human CK2-α containing the M^161^K + E^165^D mutations exhibits a ~40-fold reduction of activity towards exogenous substrates [47]. The *Tik* mutation thus acts as a CK2 dominant-negative (CK2-DN) allele, and its ability to perturb the circadian clock reflects incorporation into and downregulation of the α2β2 holoenzyme [114]. In addition to the M^161^K + E^165^D mutations, the *TikR* protein harbors an in-frame deletion of nine amino acids that are invariant between Drosophila and human CK2-α (Figure 1) and localize to a highly structured region of the protein [115,116]. This deletion compromises folding and abrogates interaction with Dm-CK2-β, consequently preventing incorporation of the TikR protein into the α2β2 holoenzyme. This is supported by the identical “*effective lethal phase*” of *Tik/Tik*, *TikR/TikR* or *Tik/TikR* flies (see Figure 1). Thus, the partial reversion of the clock phenotype by the *TikR* mutation does not reflect restored CK2 activity, but instead reflects an inability of the mis-folded TikR protein to act as a CK2-DN. In line with this interpretation, over-expression of the Tik mutant (in otherwise wild-type flies) elicits defects in the clock, and perturbs development of the eye and bristles, a characteristic of impaired Notch signaling (see Table 2 and Reference [83]). A similar impairment of Notch signaling manifests upon targeted over-expression of the single mutants, CK2α-M^161^K or CK2α-E^165^D; the former acts as a CK2-DN, whereas the latter through its ability to elicit a gain in activity of the phosphatase PP2A. Tik should therefore be considered a “double hit” with respect to Notch signaling. It remains to be clarified if this applies to CK2 functions in other developmental programs. No eye/bristle defects manifest upon overexpression of TikR protein or wild-type Dm-CK2-α (Bidwai, unpublished, and see below).

Other than defects in the circadian clock, neither *Tik/+* nor *TikR/+* flies display any other developmental abnormalities (Figure 1). The normal development of *Tik/+* animals is surprising, given the DN nature of this allele and the large number of targets of this protein kinase (Table 1). These findings raise the prospect that the circadian clock is more sensitive to levels of CK2 activity, i.e., a 50% reduction in *TikR/+* animals elicits minor defects, whereas further reductions in *Tik/+* (<50%) become rate-limiting. The absence of overt phenotypes in *TikR/+* animals could be reconciled with the findings that only a few developmental processes are haplo-insufficient, and because development is often “buffered” against fluctuations in gene-dosage (see below).

Additional mutations such as *CK2-α-G703* and *CK2-α-H3091* have also been identified, but analysis of these alleles in eye/bristle development or clock functions has not been reported. It is of interest to note that the *CK2-α-H3091* allele replaces a highly conserved Asp^212^ with Asn (Figure 1). Remarkably, the first temperature-sensitive (ts) alleles of CK2, which were isolated in the laboratory of Claiborne Glover, include a D^220^N mutation in the *CKA2* gene encoding the yeast CK2-α′ subunit [117]. Asp^220^ of yeast *CKA2* corresponds to Asp^212^ in Dm-CK2-α (and Asp^214^ in Hs-CK2-α, see Figure 1), and the targeted introduction of a D^212^N mutation also engenders a ts-behavior upon Dm-CK2-α in a yeast complementation assay [118]. The D^220/212^ site resides in the C-lobe of CK2-α, is close to the active site, and points towards the core of this region, making it likely that this is a permissive site for destabilizing CK2 structure, thereby rendering the mutant protein temperature-sensitive in yeast as well as flies. Additionally, as seen in Figure 1, many of the mutations that abrogate CK2-α activity or perturb its structure appear to reside in highly structured regions. Recent advances in evolutionary statistical coupling [119] may offer a route to better understand the clustering of these mutations, and provide new insights into the evolutionary relationships between CK2-α subunits across the tree of life. Given these findings, it will be of interest to determine if *CK2-α-H3091* mutant flies display overt developmental defects (such as defects in embryogenesis, or eye/bristle development) in a temperature-sensitive manner. If so, it will provide the first bona fide ts-allele of *Dm-CK2-α*, which should enhance our ability to better define the Dm-CK2 dependent phospho-proteome, and the multitude of developmental programs that are controlled by this protein kinase. In contrast, the *CK2-α-G703* allele harbors a W^279^G mutation, affecting a residue conserved in both yeast CK2-α genes (*CKA1/CKA2* [32]) as well as Hs-CK2-α (Figure 1, and not shown); but the mechanism underlying the lethality of this allele remains unclear. The only hypomorphic allele that has been identified to date is *CK2α-MB00477 (CK2α^MB^*). This allele results from the insertion of a P-element (a transposon) in the 5′ control region. Consequently, *CK2α^MB^* is lethal when homozygous, and these animals die at the pupal-to-adult transition (see Figure 1A). Importantly, pupal lethality is also seen in *CK2α^MB^/Tik* or *CK2α^MB^/TikR* animals, confirming that *CK2α^MB^* is a new unique hypomorphic mutation in *Dm-CK2-α*. As expected, *CK2α^MB^/+* animals display normal eye development. However, unlike *Tik* or *TikR* homozygotes, which die prior to the onset of retinal development (dashed green line in Figure 1A), *CK2α^MB^* homozygotes progress normally through the third larval stage, which is a critical juncture marking the onset of retinal neurogenesis and eye development. We discuss the nature of this allele and highlight its utility to better understand the roles of CK2 in eye development later in this review (see below).

The first mutant of *Dm-CK2-β* was the *Andante* allele (see Table 2), originally identified by Ron Konopka [120,121]. This mutation, which was mapped to the 10E1-E7 region of the X-chromosome, is in close proximity to the *Dm-CK2-β* gene [102] and was characterized by the lengthening of the circadian period and locomotor activity rhythms by 1.5–2.0 h. The nature of the mutation and the affected gene remained unknown until the laboratory of F. Rob Jackson identified it as a mis-sense mutation in *Dm-CK2-β*. This mutation called *Dm-CK2-β^And^* [122] replaces M^166^I, a residue that lies in helix α-F [112], which is positioned close to the interface between CK2-α and CK2-β. Consequently, it was thought that *CK2-β^And^* is impaired for proper assembly into the α2β2 holoenzyme or destabilizes this ternary state. However, human CK2-β with the M^166^I mutation interacts with CK2-α as efficiently as wild-type CK2-β and forms the holoenzyme with normal activity [47], raising questions on its proposed relevance to the clock defects of *CK2-β^And^* flies. Consistent with these latter findings, *CK2-β^And^* flies are viable as hemizygous males or homozygous females. A second hypomorphic allele, *CK2-β^mbuP1^*, was reported by the laboratory of Thomas Raabe. Remarkably, Jauch and co-workers demonstrated that *CK2-β^mbuP1^* impairs proliferation of Kenyon cells thus affecting development of the mushroom body, a structure key to learning and memory. Their studies also identified an excision allele, *Dm-CK2-β^mbu∆A26^*, that disrupts the *CK2-β* coding region and results in embryonic lethality when homozygous. Together with earlier findings from the mouse model [123], these studies demonstrate that *CK2-β* is an essential gene in Drosophila.

It is currently unclear why loss of the *Dm-CK2-α* gene results in lethality at the first larval stage, whereas that of *Dm-CK2-β* is embryonic lethal (see Figure 1). Maternal contribution of Dm-CK2 could account for the larval lethality of *Dm-CK2-α* mutants, but how does one reconcile the earlier lethality of *Dm-CK2-β* mutants. One possibility is differential half-life of individual subunits or their mRNAs, such that Dm-CK2-β protein/transcripts have a higher turnover-rate compared to Dm-CK2-α. This issue is unlikely to be resolved by pulse-chase analysis in S2-cells, because free subunits have not been detected in this embryonic cell type [46], and factors that regulate differential turnover and/or holoenzyme assembly might well be present only in an intact developing embryo. An alternative possibility is that differential turnover is spatially and/or temporally controlled. Current technologies do not allow us to evaluate/distinguish between these possibilities. However, the development and refinement of genome editing technologies may allow the differential tagging of Dm-CK2-β protein or transcripts to resolve these issues.

## 6. Multiple Non-Redundant Variants Encoded by the Dm-CK2-β Gene

As mentioned above, the *Dm-CK2-β* coding region encodes for five distinct protein isoforms, some of which reflect distinct splicing events and are likely produced in a tissue-specific manner. In all cases, the isoforms differ only in their C-terminal tail, which becomes appended with ~15–20 amino acids unique to each isoform. In the crystal structure of the human CK2 holoenzyme (PDB code 1 JWH, [115]), the CK2-β subunit is truncated such that it lacks the penultimate 10 amino acids. Consequently, the structural constraints on the C-terminus of CK2-β are unclear. Nevertheless, the region preceding these missing residues is a well-defined helix, which does not contribute to the CK2-β/CK2-α interaction interface, but projects away from the core holoenzyme structure. Given that CK2-β subunits generally display length and sequence heterogeneity in their C-terminal tail, one might expect that these would have minimal impact on CK2 functions. To the contrary, the laboratory of Thomas Raabe has individually tested all five Dm-CK2-β variants in an exceptionally robust in vivo functional complementation assay, i.e., their ability to rescue the lethality of the *Dm-CK2-β^mbu∆A26^* mutation. Remarkably, only three out of five Dm-CK2-β isoforms (CK2-β-VIIa, -VIIb, -VIIc) rescue lethality [111], leading to the view that these alternative C-termini alter in vivo functionality of Dm-CK2-β variants (see Table 2). In addition, they also conducted tests for phenotypic outcomes of ubiquitous expression of each isoform in otherwise wild-type flies using the *tubulin* (*tub*) promoter, and find that only two Dm-CK2-β isoforms, VIIb and VIId, elicit dominant lethality. The dominant lethality of these two isoforms may reflect competition for limiting amounts of Dm-CK2-α that is available to form the holoenzyme. Together, these in vivo results are strong indicators that these variants bias the Dm-CK2-β “interactome”, perhaps by regulating target protein specificity, cellular locale, turnover rates, etc. Alternatively, these C-terminal variations may result in holoenzyme isoforms that differ in their ability to form ternary complexes. As shown by the laboratory of Roberto Battistutta, the C-terminus of CK2-β impacts the ability of CK2 tetramers to form ternary complexes, i.e., filaments [63]. In a new trimeric ring-like structure, which they call α2β2^new^, the C-terminus of CK2-β competes with ATP for binding to CK2-α, and appears to stabilize a nonproductive conformation upon insertion into the ATP-binding pocket. Additionally, they demonstrate that this interaction impairs pairing of residues in CK2-α that are critical for catalysis and are a generally conserved feature of protein kinases. If so, this could represent a novel structural basis for CK2 downregulation. Given these findings, it is likely that CK2 holoenzymes containing Dm-CK2-β isoforms with alternative C-terminal sequences differ in their propensity to form trimeric ring-like structures. The possibility thus arises that the dominant lethality of the alternative Dm-CK2-β isoforms (VIIb and VIId) revealed by the studies of Jauch and coworkers [111] may, in fact, involve aberrant in vivo regulation of Dm-CK2. It would hence be worthwhile to investigate which of the five Dm-CK2-β isoforms favor or disfavor the formation of ring-like states.

In the same study, Raabe and coworkers also tested and demonstrated that neither Dm-CK2-β′ nor *SSL/CK2-β^Tes^* rescue the lethality of the *Dm-CK2-β^mbu∆A26^* mutation, and both elicit dominant lethality upon ubiquitous expression in wild type flies (Table 2). These findings are of interest, because even though Dm-CK2-β′ and SSL/CK2-β^Tes^ are structurally similar to Dm-CK2-β, they exhibit two differences. (1) Unlike the auto-phosphorylation site in Dm-CK2-β (M^1^SSSEE), Dm-CK2-β′ harbors the motif M^1^TDSDE, whereas it is M^1^SCPRS in SSL. Consequently, Dm-CK2-β′ may resemble a constitutively phosphorylated protein, while SSL would be refractory; (2) The acidic micro-domain is also different such that the rank order of acidity is Dm-CK2-β > *SSL/CK2-β^Tes^* > Dm-CK2-β′. Given the findings of Jauch and coworkers, it will be of interest to determine if rescue of the *Dm-CK2-β^mbu∆A26^* mutation by Dm-CK2-β requires an intact auto-phosphorylation site and/or acidic micro-domain.

As of the writing of this review, no mutants of the *Dm-CK2-β′* or *SSL/CK2-β^Tes^* genes are available, precluding predictions of their biological functions. In a yeast-based assay, these two proteins appear to partially compensate for phenotypes elicited by loss of the yeast CK2-β genes [107], but this may not be an appropriate assay for in vivo functions. However, considering their tissue-specificity and/or male-specificity and dominant lethality, it is reasonable to speculate that if these isoforms were to downregulate CK2 activity, alter its target specificity, or impact formation of trimeric rings, these functions may be tied to male development. Nevertheless, the studies of the Raabe laboratory make it likely that the diverse CK2-β like proteins in Drosophila (splice variations and distinct genes) serve unique tissue, developmental stage, or sex-specific functions or confer unique regulation upon the enzyme itself. To our knowledge, this level of complexity in CK2-β subunits has not been described for any metazoan organism, but is not without precedence. For example, the laboratory of Marc Vidal has reported that the “interactome” of a protein is significantly altered by splicing variations, almost as if the alternative products are encoded by distinct genes [124]. It would hence be worthwhile to determine the extent of overlap and non-overlap of proteins that interact with alternative isoforms that are encoded by the Dm-CK2-β gene versus those that interact with *Dm-CK2-β′* or *SSL/CK2-β^Tes^*.

## 7. The DPiM Database Provides New Insights into the Dm-CK2 Interactome and Subunit Specific Interactions

Whole cell proteomics affords an unbiased route to identify interacting partners. Such an approach has been taken using Drosophila S2 cells [72,73]. In this comprehensive study, Guruharsha and coworkers expressed ≥5000 individual FLAG-HA epitope-tagged Drosophila proteins, which was followed by co-affinity purification coupled to mass spectrometry analysis. This study has enabled the determination of a vast number of protein complexes, which they call the ‘Drosophila protein interaction map (DPiM, https://interfly.med.harvard.edu). Given the structural and functional diversity of CK2-β isoforms, we analyzed the DPiM database for all five Dm-CK2 subunits, i.e., CK2-α, CK2-β, CK2-β′, Stellate, and SSL/CK2-β^Tes^ (see Table 3). Although the DPiM database includes analysis of only three, CK2-α, CK2-β, CK2-β′, new insights nevertheless emerge.

### 7.1. Interactions between Dm-CK2 Subunits

As expected (see Table 3), these studies identified the canonical interactions between CK2-α + CK2-β and CK2-α + CK2-β′, consistent with previous studies using the yeast two hybrid approach [101,106,125]. Given the tetrameric structure of the human/Drosophila CK2 holoenzyme, the co-purification of CK2-α using FLAG-HA-CK2-α is unlikely to reflect direct interactions, but instead “bridged” by CK2-β. Surprisingly, even though S2-cells endogenously express both CK2-β and CK2-β′, the only interactions that were detected were CK2-β + CK2-β and CK2-β′ + CK2-β′ (Table 3). The absence of cross-CK2-β subunit interactions in S2 cells suggests that the CK2-holoenzyme cannot be generated using mixed CK2-β dimers, such as CK2-β + CK2-β′. Whether this also applies to the Stellate, and SSL/CK2-β^Tes^ proteins remains unknown. The CK2-β-vs.-CK2-β′ subunit-specific bias (for dimerization) makes it likely that sector analysis [126,127] may provide new insights into the evolution and diversity of the CK2-β family in Drosophila. The possibility that this aspect of CK2 structure impacts in vivo functionality is exciting, and may represent a unique mode of regulation, which has remained the most elusive aspect of CK2 functions across all eukaryotes.

### 7.2. Dm-CK2-α Interactors

In large part, the proteins that interact with Dm-CK2-α are unique, and encompass a multitude of functions. Notably absent from this list are proteins whose targeting by CK2 has been confirmed by combined biochemical and genetic studies (see Table 1). The only proteins for which such evidence exists are Topo-II and FMR1. Interestingly, four of the interacting proteins, Dek, Dre4, Scf, and Ssrp, were also identified as interactors of Dm-CK2-β (see below). It is of interest that the DPiM database reveals interactions of CK2-α with Mts, the catalytic subunit of the phosphatase PP2A [128]. This phosphatase plays multiple essential roles in metazoan cell/organismal biology; it is a known tumor suppressor that is involved in oncogenesis [129,130], and regulates the Notch and Hedgehog signaling pathways [131,132,133], autophagy [134] and the cell-cycle [133,135]. Consequently, loss of Mts activity is lethal in Drosophila. Of interest, the CK2-α + PP2A interaction was originally reported for the human proteins, and occurs via the M^161^IDHE^165^NRKL motif [136], also present in the oncogenic virus SV-40. Intriguingly, this very motif is mutated in the *Dm-CK2-α* allele *Tik* (see Figure 1), and functional studies in Drosophila reveal that the CK2α-E^165^D mutation elicits phenotypes that mimic overexpression of PP2A-Mts or its regulatory subunit PP2A-Widerborst [114,131]. These studies make it likely that the CK2-PP2A interaction serves to downregulate phosphatase activity. The remaining proteins fall into four classes; chromatin organization, RNA binding proteins, transcription factors, and others regulating signaling and the cytoskeleton. It will be of interest to determine if these proteins are targets of CK2 in vitro, and the consequences of phosphorylation during development.

Interactors of Dm-CK2-β and Dm-CK2-β′ are listed in Table 4, and are sorted by those that are unique to each subunit and those that overlap. In each case, the DPiM database includes several ribosomal proteins. Although these interactions may involve regulation of “*sentinel-like*” functions of ribosomal proteins [137], we have not considered this class of proteins as bona fide CK2 interactors because they often appear in large scale proteomic and yeast two-hybrid screens and can represent potential artefacts.

### 7.3. Interactors of Dm-CK2-β

Unique interactors of Dm-CK2-β encompass proteins involved in protein folding and turnover, chromatin structure and transcriptional control, cytoskeleton, cell cycle progression, RNA-processing, and neuronal development. Remarkably, only, dMi-2, a protein involved in nucleosome remodeling, has also been identified as a target for CK2 in genetic studies (see Table 1 and Table 4). In contrast, four interactors of Dm-CK2-β, i.e., Dek, Dre-4, Ssrp and Scf, have also been isolated as interactors of Dm-CK2-α (see Table 3). It will be of interest to determine if these four proteins directly interact with Dm-CK2-α or Dm-CK2-β, or represent proteins that interact with the reconstituted holoenzyme in S2 cells. The remaining proteins interact exclusively with Dm-CK2-β, consistent with the view that some targets of CK2 such as Hairy [90], Raf [138], etc., can interact only through interaction with this regulatory subunit.

### 7.4. Interactors of Dm-CK2-β′

The interactors of Dm-CK2-β′ are not as extensive as those for Dm-CK2-β (Table 4), and include regulators of signaling, chromatin structure, DNA-replication/damage-response, etc. It is somewhat unexpected that proteins that interact with Dm-CK2-β and Dm-CK2-β′ do not overlap extensively. This preferential interaction partner specificity may underlie the ability of Dm-CK2-β′ to elicit dominant lethality upon ubiquitous expression (see above), whereby the inappropriate (spatial and/or temporal) presence of this subunit results in the formation of the CK2-β′-containing holoenzyme (α2β′2) due to competition for a common pool of CK2-α. Given the distinct “interactome”, this alteration in the CK2 holoenzyme could diminish phosphorylation of proteins by the endogenous α2β2 holoenzyme or redirect activity to substrates incompatible with normal cellular and organismal viability.

### 7.5. Interactors Shared between Dm-CK2-β and -β′

Of the interacting proteins identified in the DPiM database, only four (Fax, Elongin-B, Otefin and Nopp140) are shared between Dm-CK2-β and -β′. Of interest is Nopp140 (see Table 4). Nopp140 is a highly conserved phosphoprotein that shuttles cargo between the nucleolus and the cytosol [139], and is thought to be critical in proliferative cells for Cajal body functions. While this protein has a native mass of ~70 kDa (as predicted from its gene structure), it appears as a 140 kDa polypeptide, primarily due to CK2-dependent phosphorylation. It has been estimated that Nopp140 may be phosphorylated at ≥70 sites within its C-terminal region, which is rich in Ser and Asp residues, and is conserved between Drosophila and mammals. These modifications, which confer a highly negative charge to the C-terminal domain, appear necessary for in vivo functioning of Nopp140, i.e., binding cargo proteins that contain nuclear localization signals (NLS). Interestingly, Nopp140 was not identified in the DPiM database as an interactor of Dm-CK2α (see Table 3), suggesting that CK2 targets this protein in a β/β′-dependent manner. Its identification as an interactor of both Dm-CK2-β and Dm-CK2-β′ strongly suggests that Nopp140 is modified by CK2 in all cells regardless of the expression patterns of these two CK2-β homologues, which would not be surprising given cell autonomous roles for this phospho-protein, and because loss of Nopp140 is cell lethal.

Together, the interactors of CK2 identified through combined biochemical/genetic approaches and the DPiM database reveal that our current knowledge of the Dm-CK2 “interactome” is, at best, partial, and the absence of developmentally important/relevant targets suggests that a case-by-case approach may still be required to identify its targets in Drosophila.

## 8. Roles of CK2 in Drosophila Eye Development

The functions of CK2 in other developmental programs have been inferred from tissue-specific and ectopic overexpression of the CK2-DN (Tik) mutant protein, RNAi against CK2 subunits in otherwise wild-type flies, or upon expression of CK2 target proteins harboring Ala/Asp mutations at known sites for phosphorylation. This has been achieved in large part due to the availability of the tissue-specific binary Gal4-UAS system [140,141,142], which enables genotype/phenotype relationships to be evaluated with mutants that would otherwise be dominantly lethal. Using this approach, it has been found that CK2 plays an important role in Notch signaling during development of two sensory organs (eye and bristle), through its targeting of the bHLH transcription factors E(spl)-M8, -M7, and -M5 [84], which are terminal effectors of this pathway [143,144,145]. In the following section, we review key findings on the roles of CK2 in eye development with an emphasis on its functions in early retinal neurogenesis, the potential implications on early neurogenesis in the mammalian retina, emerging data supporting the likelihood that this protein kinase plays additional roles in the developing eye, and conclude with a review of potentially new targets for this protein kinase.

Studies from our laboratory are revealing the importance of CK2 to the Notch signaling pathway during Drosophila eye development, with direct implications for a similar process in mammals, i.e., the specification of the Retinal Ganglion Cells (RGCs). Here, we focus on the Notch pathway in Drosophila, a preeminent genetic model that has been instrumental in the identification of the core components and regulators of this pathway, and has laid the foundations for our understanding of the mechanisms of Notch signaling [146,147,148,149,150], its importance to the development of other animals [148,151] and its association with disease states [152,153,154,155,156]. Although Notch signaling regulates diverse developmental programs, studies from our laboratory have primarily focused on its regulation by CK2 in early eye development.

The Drosophila eye has served as a model for understanding cell proliferation, cell signaling, polarity, specification and differentiation [157,158,159]. The compound eye of Drosophila is composed of ~750 units called ommatidia (also known as facets) which are patterned in a precise pseudo-crystalline array. Each facet is composed of eight photoreceptor neurons (Retinula cells, R1–R8), 12 accessory non-neuronal cells, and one sensory inter-ommatidial bristle (IOB). The precise hexagonal geometry of the adult eye and its constituent cell types are both essential for proper visual perception, reasons for which these features are highly conserved across all insects.

**Figure 2:**
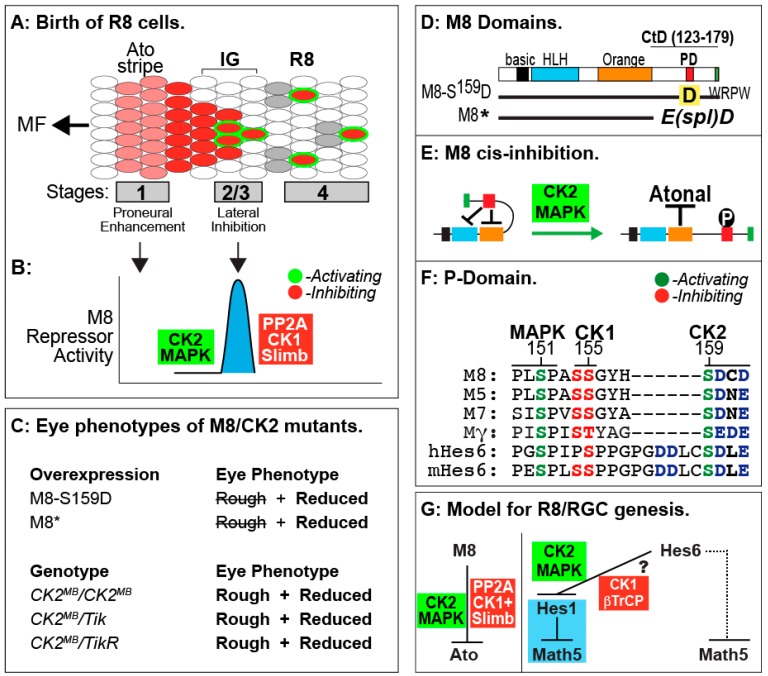
(**A**) R8 birth; Ato (pink/red), Sens (blue) and secondary R cells (grey); (**B**) CK2 and Mitogen Activated Protein Kinase (MAPK) activate M8 at stage-2/3 of the MF, and after R8 selection, PP2A and CK1 + Slimb mediate inactivation and/or destruction; (**C**) Eye phenotypes of CK2 and M8 mutants; (**D**) Functional domains in M8, and location of the phosphorylation domain (PD) in the C-terminal domain (CtD). The WRPW motif in M8 binds the essential co-repressor Groucho, and M8* (the product of the *E(spl)D* mutation) eliminates the CtD; (**E**) Regulation of cis-inhibited M8 by CK2 and MAPK. Note that in cis-inhibited M8, the PD blocks either HLH (blue) or Orange domains preventing interaction with Atonal; (**F**) Conservation of the PD in Drosophila M8/M5/M7/Mγ and human/mouse Hes6; (**G**) Model for CK2 regulation of Drosophila M8 and mammalian Hes6. Dotted line denotes non-canonical mode of Hes6 action.

Eye development initiates during the third larval stage (see Figure 2), and involves progressive stages of cell specification and morphogenesis of a monolayer neuro-epithelium called the eye/antennal disc (eye anlagen). This onset of retinal neurogenesis is marked by the specification of the first photoreceptors, the R8 cells, and occurs in a wave of cell specification called the Morphogenetic Furrow (MF, see Figure 2A). In contrast to the specification of the R8 cells in the MF, recruitment of all secondary photoreceptors (R1–R7) occurs posterior to it. The MF therefore represents a 48-hour window of development that covers all retinal neurogenesis. For a more detailed description of R8 cell specification and roles in this sensory organ, see Reference [160]. In Drosophila, the bHLH transcription factor Atonal (Ato) specifies the R8 photoreceptors [161], which subsequently recruit all later retinal cell types’ characteristic of the ommatidium (see above). The R8s are not clonally derived, and each is the outcome of inductive recruitment that occurs in a precise spatial/temporal manner. Hence, in the absence of Ato, no R8s or other retinal cells form, thus ablating the eye. Likewise, a defect in human *Atoh7* elicits blindness due to a loss of RGCs and the optic nerve [162], suggesting that lessons learned from fruit flies are applicable to mammals. R8/RGC patterning demands controlled repression of Ato/Atoh7 by the Enhancer of split (E(spl)) proteins (called Hes in vertebrates [163,164]), which also pattern other tissues and whose regulation by CK2 is now well understood (see below). Hence, the regulation of E(spl) activity during R8 patterning is broadly applicable and relevant to understanding Notch-dependent human developmental disorders.

During R8 ontogeny, Ato expression initiates as a stripe at the leading edge (stage-1) of the MF (Figure 2A). Cell-autonomous *ato* auto-activation [165] then produces pre-R8 cell clusters [166], the intermediate groups (IGs, see Figure 2A). Notch signaling through E(spl) proteins then acts to repress Ato (non-autonomously) at stage-2/3 in all but one cell from each IG [167]. That cell continues to maintain *senseless* (*sens*) expression and differentiates as an R8 [168,169]. The other cells of each IG remain in a non-neural (undifferentiated) state. This binary cell fate determination is termed lateral inhibition, and functions similarly during bristle patterning [170,171]. Repression of Ato by E(spl) proteins is therefore key to generating patterned R8s posterior to the MF (Figure 2A). One unusual aspect of Notch signaling during R8 ontogeny is that Notch is necessary for proper expression of the proneural protein Ato at stage-1, as well as that of the E(spl) repressors at stage-2/3 [172]. Given that the MF is a moving wave, cells at stage-1 are separated from those at stage-2/3 by ~5–10 min [173]. How Notch achieves these two mutually antagonistic functions within this short time frame remains unclear. In canonical Notch signaling, receptor activation elicits cleavage of the Notch intracellular domain (NICD) and its translocation to the nucleus to effect target gene activation [174]. It is difficult to envision how this mode of gene regulation rapidly switches from proneural to repressive modes. Our work (see below) suggests that CK2 plays a crucial role in enforcing a short temporal delay such that the repressive effects of Notch only manifest at stage-2/3 of the MF (Figure 2B). In a sense, CK2 may thus “decouple” these two phases of Notch signaling.

As mentioned above, E(spl) protein expression is necessary to resolve a single R8 from each IG. The *E(spl)Complex* encodes seven homologous and highly conserved bHLH repressors [144,145], of which M8, Mγ and Mδ are expressed in the MF [175]. While loss of the *E(spl)C* elicits the abnormal specification of extra (“twinned”) R8s from an IG [167], over-expression of M8/Mγ/Mδ (at stage-2/3) does not dominantly repress Ato or the R8 fate [172,176,177]. The importance of M8 in Ato repression is highlighted by the *E(spl)D* mutation. This mutation, serendipitously identified in the 1950s [178,179], encodes M8* lacking its C-terminal domain (CtD, Figure 2C,D) and potently represses Ato, thus ablating R8s and the eye [177,180]. The mechanism underlying the hyperactivity of M8* remained an enigma, until we discovered that the E(spl) proteins are targeted by CK2 [84]. Specifically, CK2 interacts with and phosphorylates E(spl)-M8, -M7 and -M5; this modification targets a highly conserved phosphorylation domain (PD) located within the CtD (see Figure 2D,F). Remarkably, replacement of the CK2 phosphoacceptor of M8, Ser^159^, with Asp (Figure 2D) results in a variant that ablates eye development via a mechanism virtually identical to that of M8* (Figure 2C, and see [84]). Phosphorylation displaces the autoinhibitory CtD, to expose the Orange domain and permit binding and repression of Ato (Figure 2E), a regulation that is circumvented by CtD deletion in M8* [181]. Consistent with a role in M8 repression of Ato, reduced CK2 activity elicits “twinned” R8s [182], as occurs upon excessive expression of Ato [183] or upon loss of *E(spl)C* [167]. These findings thus demonstrate that CK2 is a key participant of Notch signaling at the onset of eye development.

More recent studies are revealing that regulation of M8 activity involves multi-site PTM of the P-domain, and this involves activation of M8 by the kinases CK2 + MAPK [184], whereas inactivation involves the phosphatase PP2A, either alone or in combination with CK1 plus Slimb (βTrCP, see Figure 2B). Such a model posits that controlled activation and inactivation ensures that repressive effects of M8 occur in a spatially and/or temporally controlled manner. This mode of regulation could enable Notch to signal in a “pulsatile” manner, akin to that during genesis of somites. We believe that this mode of signal regulation may have direct implications on birth of RGCs in mammals, where Math5 (murine Ato homolog 5) expressivity is refined through Hes repressors (the homologues of fly E(spl) proteins). Specifically, previous studies have demonstrated that Hes6 (the homologue of fly M8) harbors a highly conserved P-domain that is targeted by CK2 and MAPK [185,186], and conserves sites for CK1 and βTrCP (Figure 2F). Furthermore, CK2 phosphorylation of mouse Hes6 also mediates its interaction with Hes1, the repressor of Math5/Atoh7 [187,188,189,190]. The role of Hes6 phosphorylation during RGC birth remains unknown; a possible model is discussed in the next section.

## 9. Lessons from Drosophila R8 Cells Applied to Birth of Mammalian RGCs

The striking parallels between the fly R8 and mammalian RGC [191], and the conserved P-domain (Figure 2F,G) begs the question of the role(s) of CK2, CK1 and βTrCP in the regulation of Hes6. Like the R8 cell, RGC patterning requires repression of Math5/Atoh7 by Hes1 [189,190,192,193,194]. This activity of Hes1 is, in turn, antagonized by Hes6 through protein–protein interactions, which (in cultured neuronal cells) requires phosphorylation of Hes6 by CK2 [187]. Post-translational regulation of Hes6 may have two distinct outcomes (Figure 2G). In the canonical mode (Figure 2G, blue box), CK2 would promote Hes6 inhibition of Hes1, thereby favoring Math5 activity and the RGC fate. In contrast, CK1 + βTrCP would promote Hes6 degradation, thereby permitting Hes1 to repress Math5 and the RGC fate. If so, the signaling circuit in the mammalian retina would be the inverse of that in flies. In a less likely alternative, Hes6 directly antagonizes Math5. If so, CK2 would promote Hes6 inhibition of Math5 and the RGC fate, while CK1 + βTrCP would promote, a mechanism akin to that in flies. However, this latter model would require that Hes6 has altered partner preference during RGC patterning. We predict that the expression of normal and mutant Hes6 (refractory to or mimicking CK2, MAPK, CK1 or βTrCP sites) in the early embryonic mouse retina may resolve the role and regulation of Hes6 during RGC birth and which of these two models is correct. In addition to the retina, post-translational regulation of Hes6 may occur elsewhere, given that Hes6 plays roles in late embryogenesis, myogenesis [195,196,197], and postnatal development [198,199], and its overexpression is linked to gliomas and breast and prostate cancer [200,201,202]. Our findings in Drosophila may aid efforts to answer similar questions in the more complex mouse model, and determine if mis-regulated Hes6 activity is linked to disease states.

## 10. Additional Roles of CK2 during Drosophila Eye Development

Unlike the role of CK2 during (Notch-dependent) birth of R8 cells, its roles later in eye development, i.e., the recruitment of R1–R7 photoreceptors have not been forthcoming. For example, after birth of R8s, Notch signaling is required in a reiterative manner for the specification of the R1–R7 cells, which are born in a precise order. These include, in order, the R2/R5, R3/R4, R1/R6 followed by the R7 cell. The hypomorphic allele of *Dm-CK2-α* called *CK2α-MB00477* (abbreviated as *CK2α^MB^*, see Table 2) is the first mutation that directly implicates roles for CK2 at multiple stages of eye development. Importantly, this mutation provides “forward” genetic evidence, which is generally considered to be a benchmark in the field of Drosophila genetics. Our analyses of this mutation (Figure 1A) reveal that *CK2^MB^* homozygous animals, or those that are trans-heterozygous with *Tik* or *TikR*, complete the third larval stage and die at the mid-pupal transition. As these animals are competent to transition through these two critical stages of development (relative to the onset of retinal neurogenesis, see Figure 1A), the compound eyes can be evaluated (for patterning and size) following their dissection from the pupal case. These animals display a highly perturbed eye, which is both rough in appearance and significantly (~50%) reduced in size (Figure 2C), and these defects are completely rescued by expression of a *tub-Dm-CK2-α* construct (Bandyopadhyay and Bidwai, in preparation). The former (rough eye) phenotype results from impaired lateral inhibition, i.e., the defective refinement of a single R8 from an IG. On its own, a defect in lateral inhibition cannot account for the reduced eye field, raising the likelihood that this phenotype may arise from defective recruitment of secondary photoreceptors due to defective (reduced) phosphorylation of additional CK2 targets in the developing eye.

## 11. Potential CK2 Targets Identified Via the Transcriptome of the Developing Eye

The complex eye defects of *CK2^MB^*/*CK2^MB^* homozygous animals raise the prospect that CK2 plays additional roles in eye development, beyond that required for controlling R8 patterning through its regulation of E(spl)-M8 (see above). To reveal eye-specific proteins that may be regulated by CK2, we analyzed the “transcriptome” of the developing eye disc to identify additional targets of CK2. This sequence based prediction for high likelihood CK2 targets is facilitated by earlier studies that defined the substrate specificity of CK2, the consensus recognition, and the impact of amino acids vicinal to the phospho-acceptor (Ser/Thr). The following general principles have emerged for CK2; (1) it is an acidophilic protein kinase; (2) the substrate specificity is best described as S/T-E/D-x-D/E; (3) the presence of additional Asp/Glu residues either N- or C-terminal to the phosphoacceptor(s) further enhances phosphorylation of target proteins; (4) phosphorylation is negatively impacted by basic residues such as Arg/Lys since the presence of these residues within the consensus abrogates targeting by CK2; and (5) the presence of phospho-Ser/Thr (pSer/pThr) C-terminal to the primary phosphoacceptor augments targeting by CK2, revealing that this enzyme can participate in hierarchical phosphorylation either alone or in concert with other protein kinases. It should, however, be noted that some sites may be solvent exposed, but inaccessible in the tertiary structure, so this approach is, at best, predictive.

Eye disc specific gene expression patterns were identified from Flybase (flybase.org), and queried for proteins predicted to harbor CK2 consensus sites. Although protein abundance, regions of intrinsic disorder and stoichiometry appear to correlate with phospho-site conservation [203,204,205], we did not incorporate these principles into our analyses, because much of this information is still lacking for the proteome of the developing eye anlagen. The first round of analysis only included sequences from *D. melanogaster*, and this identified ~180 genes. Subsequently, homologous proteins were identified from 11 other fully sequenced and annotated Drosophila species (flybase.org) that encompass ~60 × 10^6^ years of evolution [206,207], with the expectation that only high probability CK2 target sites with functional importance should be resilient through this time frame. The list of potential targets (Table 5) therefore only includes homologous proteins in which the CK2 site is conserved in all 12 species.

As is evident from Table 5, this list of high likelihood targets of CK2 includes 58 proteins regulating diverse aspects of eye development. Here, we review these proteins in accordance with their structural classification, biochemical functions and mutant phenotypes.

### 11.1. Transcription Factors

Of the ~60 interactors, >30% (21 out of 58) represent transcription factors or proteins that impact gene expression through modification of chromatin structure. Notably, two of these, e.g., Eyeless (Ey) and Eyegone (Eyg), are transcription factors that lie at the apex of the retinal neurogenesis hierarchy, a class of proteins called the “retinal determination” (RD) genes (for reviews, see [208,209,210]). This view reflects the remarkable findings from the laboratory of the late Walter Gehring (Basel) that ectopic expression of the Ey gene induces the formation of ectopic eyes in non-retinal tissues such as the wings, legs, and antenna [211]. This remarkable ability reflects the fact that Ey induces the transcription of additional RD-genes such as Sine Oculis (SO) and Eyes Absent (Eya) [212,213,214]. Both SO and Eya are predicted to be targets of CK2 (see Table 5), and studies from our laboratory have now confirmed this to be the case for SO (Majot and Bidwai, unpublished). The possibility that CK2 may regulate four critical eye-determination transcription factors (Ey, Eyg, SO and Eya) may underlie the complex eye defects of *CK2^MB^*/*CK2^MB^* animals (Figure 2C). The potential targeting of Eyg is an excellent example for evaluating the emergence of orthologues. The Drosophila genome encodes two such genes, *Eyg* and *Twin of Eyegone* (*Toe*). Eyg harbors the CK2 consensus site D**S**DEEINV (bold residue is likely to be targeted by CK2), whereas the corresponding site on Toe is E**E**EEVINV. Because the D/E residues mimic Ser^P04^, it raises the possibility that Eyg is CK2-regulated whereas Toe is CK2-independent. It will therefore be of interest to determine if Eyg is modified by CK2, identify the site(s) of phosphorylation, and determine the in vivo activities using its ability to elicit ectopic eye formation. Conversely, it would also be of interest to conduct domain swapping experiments to determine if Toe can be rendered CK2-dependent. Such studies could better illuminate the relevance of CK2 to these critical eye-specific TFs.

### 11.2. Signaling Pathways

Cell signaling is vital for most developmental programs. Two important and intersecting pathways are Notch and EGFR. While our previous work has revealed the centrality of CK2 to Notch pathway output in the developing eye and bristle (Section 8, see above), the role of this kinase in EGFR/Sevenless (Sev) signaling has not heretofore been suspect. While CK2 regulation of Raf, a mediator of EGFR/Sev signaling in flies is known [53] and shares many similarities to that in mammals, if/how Raf phosphorylation fine-tunes signaling has remained obscure. In addition to EGFR itself, CK2 consensus sites are conserved in several components regulating this pathway such as Asteroid, Daughter-of-Sevenless, Ret, and Sina, across ~60 × 10^6^ years of Drosophila evolution. While Notch and EGFR have been generally thought to play opposing roles during development [215,216], our findings that EGFR/MAPK signaling is necessary for activity of the Notch effector E(spl)-M8 (Section 8, see above) make it likely that the former proposal is an oversimplification. If CK2 were to stimulate EGFR/MAPK signaling, it would place CK2 at the heart of the repressive effects of Notch signaling, without which R8 photoreceptors could not be patterned in the developing eye, which would perturb all subsequent steps of retinal neurogenesis, adult eye architecture, and vision. Given this possibility, a full and proper biochemical and genetic investigation of these RTK signaling components seems warranted.

### 11.3. Protein Turnover

Controlled protein turnover lies at the heart of eye development, wherein controlled degradation of transcription factors such as E(spl)-M8 seems necessary to allow timely termination of Notch signaling. We think that degradation, in principle, could be broadly applicable to all aspects of Notch signaling. Among the proteins identified as potential targets of CK2 are Neuralized (Neur), Fat-Facets (Faf), and Liquid-Facets (Lqf). Neur, a member of the RING family E3 ubiquitin ligase, is a key component of Notch signaling pathway, wherein it regulates activation of the Notch ligand via endocytosis [217,218]. In contrast, Faf is a de-ubiquitination (DUB) enzyme that negatively regulates RTK/Ras/MAPK signaling [219,220], whereas Lqf possesses Ubiquitin-interaction motifs [221]. Mutations in all three, Neur, Faf and Lqf, are associated with defects in eye development, and thus it will be of interest to determine how CK2 phosphorylation regulates these three proteins.

### 11.4. Regulators of the Cell Cycle, Cell Death, Cell Polarity and Cytoskeleton

Eye development hinges upon controlled proliferation of the eye anlagen, coordination of planar cell polarity that regulates photoreceptor positioning, cell death to remove excess non-specified and non-differentiated cells, and regulation of the cytoskeleton, which is necessary for formation of the MF, the apico-basal constriction that marks the initiation of retinal neurogenesis. Members controlling each of these aspects appear in the list of putative, highly conserved, targets for CK2. These include, proteins regulating the cell cycle such as the CDK inhibitor Decapo (Dap), the ATR-Chk1 checkpoint pathway component Claspin, and AXIN1 upregulated 1 (Axud1), and the well-known regulator of apoptosis Head involution defective (Hid). Other proteins regulating the cytoskeleton include Terribly reduced optic lobe (Trol), and Prickle, a key regulator of planar cell polarity in the developing eye.

Given the diverse array of potentially new targets of CK2 and the complex eye defects of *CK2^MB^* flies, a proper investigation of the proteome of the developing eye that is targeted by this kinase is warranted. Such studies will not only illuminate the extent to which CK2 regulates retinal neurogenesis in flies, but may also reveal new insights into its roles in mammalian eye development.

## 12. Summary and Future Perspectives

Many aspects of CK2 functions in Drosophila have emerged from studies from several laboratories. These include mutants, subunit diversity, functional non-redundancy of CK2-β homologues, interacting proteins and, importantly, the diverse roles played by this protein kinase during development. Many of these processes are likely to represent conserved features of animal development. The challenge before us is to decipher which roles are universally applicable, and which are specific to taxonomic groups. The availability of CK2 mutants with a perturbed eye now enables the application of phospho-proteomic studies, such as through the use of Phos-Tag to identify all phosphoproteins in the developing eye disc [222], rescue *CK2^MB^*/*CK2^MB^* homozygous animals with a CK2α-Apex fusion [223,224] enabling direct identification of the CK2 “interactome”, and finally through genome editing of individual CK2 target genes. Such studies, will, in due course, reveal the development-specific targets of DmCK2, and provide insights and routes to similar analysis in other vertebrate model organisms.

## Figures and Tables

**Table 1:** CK2 targets from FlyBase (November 2016).

Protein	Function	Effect of Phosphorylation
Ankyrin-2	Cytoskeletal Adaptor	Maintenance of synaptic stability
Antennapedia	Transcription Factor	Spatial restriction of activity
Armadillo	Transcription Factor	Phosphorylation triggers degradation
Cactus	Transcription Factor	Required for activity during axis formation
Clock	Transcription Factor	Stabilizes Clock
CREB2	Transcription Factor	Inhibits DNA binding
Dishevelled (dsh)	Transcription Factor	Influences Wg/Wnt signaling
dMi-2 (DPIM)	Chromatin Structure	Inhibits nucleosome-stimulated ATPase
E(spl)-M8/M5/M7	Transcription Factor	Phosphorylation required for repressor activity
Engrailed	Transcription Factor	Phosphorylation enhances DNA-binding
Enhancer of Rudimentary	Transcription Factor	Promotes and inhibits activity
FMR1 (DPIM)	RNA-Binding	Affects dimerization and RNA-binding
GAGA factor (519)	Transcription Factor	Reduced DNA binding affinity
Groucho	Transcription Factor	Stimulates short range repression
Hairy	Transcription Factor	Promotes repressor activity
Heterochromatin protein HP1	Chromatin Structure	Stimulates DNA binding
mushroom body miniature	Ribosome Biogenesis	Promotes nucleolar localization
NAP1	Chromatin Structure	Affects degradation and cellular locale
Odd	Transcription Factor	Inhibits Groucho binding and repression
Orb (CPEB-family)	RNA-Binding	Promotes Orb activity
P element Somatic Inhibitor (PSI)	Splicing factor	Modulates interactions with splicing factors
Period	Circadian Clock	Promotes nuclear entry
Raf	Protein Kinase	Required for ERK activation
Ribosomal S6 kinase	Protein Kinase	Required for activity
RPL-22	Ribosome Structure	Unknown
Smoothened	Signaling	Stabilizes and promotes Hedgehog signaling
Syntaxin-1	Membrane Protein	Stimulates interaction with Dopamine Transporter
Timeless	Circadian Clock	Promotes nuclear entry
Topoisomerase II (DPIM)	DNA-replication	Stimulates activity
Warts	Protein Kinase	Indirectly promotes Warts suppression of Yorkie

DPiM (in red) denotes proteins identified in the Drosophila Protein Interaction Map.

**Table 2:** Subunit diversity and non-redundancy of Dm-CK2β isoforms.

**Genes Encoding CK2 Subunits in Drosophila**			
**Gene (Chromosome)**	**Isoforms**	**Alleles**	**Nature**
*CK2α* (III)	Single	*CK2α-Tik*	M161K + E165D
		*CK2α-TikR*	Loss of Function
		*CK2α-MB00477*	Hypomorphic
		*CK2α-G703*	W279G
		*CK2α-H3091*	D212N
*CK2β* (X)	Multiple	*CK2βAndante*	M166I
		*CK2βmbuP1*	Hypomorphic
		*CK2βmbuΔA26-2L*	Loss of Function
*Stellate* (X)	Single	None	N/A
*CK2β′* (II)	Single	None	N/A
*SSL/CK2-βTes* (II)	Single	None	N/A
**Phenotypes of Ectopic Expression**			
**Isoform**	**Tissue**	**Overexpression Phenotype**	
CK2α-WT	Proneural cluster	No Effect	
CK2α-Tik	Proneural cluster	Impaired Notch Signaling (eye & bristle)	
CK2α-M161K	Proneural cluster	Impaired Notch Signaling (eye & bristle)	
CK2α-E165D	Proneural cluster	Impaired Notch Signaling (eye & bristle)	
CK2β-VII-a	Ubiquitous	No Effect	**Rescues *CK2β^∆A26^***
CK2β-VII-b	Ubiquitous	Dominant Lethal	**Rescues *CK2β^∆A26^***
CK2β-VII-c	Ubiquitous	No Effect	**Rescues *CK2β^∆A26^***
CK2β-VII-d	Ubiquitous	Dominant Lethal	No rescue of *CK2β^∆A26^*
CK2β-VII-d-VI	Ubiquitous	No Effect	No rescue of *CK2β^∆A26^*
Stellate	Not Tested	N/A	Not tested
CK2β′	Ubiquitous	Dominant Lethal	No rescue of *CK2β^∆A26^*
SSL/CK2-βTes	Ubiquitous	Dominant Lethal	No rescue of *CK2β^∆A26^*

Yellow box highlights ability to complement the lethality of *CK2β^∆A26^* mutants. CK2-β isoforms that fail to rescue loss of the X-linked CK2-β gene are indicated in red.

**Table 3:** The DPiM database reveals novel insights into the Dm-CK2 interactome.

**Dm-CK2-α Interactors in Drosophila S2-Cells (DPiM)**			
**Protein**	**Function**	**Protein**	**Function**
CK2-α	CK2 Catalytic Subunit	Lasp	Cytoskeletal organization
CK2-β	CK2 Regulatory Subunit	Rump	RNA-binding
AGO2	RNA-binding	Ran	Small GTPase
pAbp	Poly-A binding protein	Rack1	Receptor for activated PKC
glo	mRNA binding	Topo2 (Genetic)	DNA Topoisomerase 2
FMR1 (Genetic)	Fragile-X syndrome	p38b	MAP-kinase
Dek (CK2-β)	Homeodomain	Wmd	Wing morphogenesis
Rasputin	RNA-binding	Mts	PP2A catalytic subunit
Dre4 (CK2-β)	Chromatin-binding	Scf (CK2-β)	Chromatin Organization
Vig2	RNA-binding	Su-var(3)9	Chromatin regulator
Ssrp	HMG Box Domain	14-3-3	pSer binding
**Interactions between Dm-CK2-α, Dm-CK2-β and Dm-CK2-β′**			
**FLAG-HA-Fusion (Bait)**	**Interacting Proteins**	**Interactions Not Detected**	
CK2-α	CK2-α, CK2-β	CK2-β′	
CK2-β	CK2-α, CK2-β	CK2-β′	
CK2-β′	CK2-α, CK2-β′	CK2-β	

Interactions shared with CK2-β in the DPiM database or those revealed by genetic studies are highlighted in red.

**Table 4:** Dm-CK2-β and Dm-CK2-β′ interactors in Drosophila S2-cells (DPiM).

**Interacting Partners Unique to Dm-CK2-β**			
**Protein**	**Function**	**Protein**	**Function**
Dek (CK2-α)	Homeodomain	dMi-2 (Genetic)	Nucleosome binding
CG13800	Actin-Binding	Tango7	Neuron morphogenesis
Dre4 (CK2-α)	Chromatin Binding	CG3817	rRNA processing
Ssrp (CK2-α)	HMG Box Domain	CG1677	Zinc Finger Protein
eIF-3-S8	Translation Factor	CG5525	HSP60-family
CDK12	Protein Kinase	Xpc/mus210	Xeroderma pigmentosum-C
CG7033	HSP60-family	Cpb (CG17158)	Actin Capping Protein
CG8258	Unknown	Prp38	pre-mRNA processing
Arp14D	Actin related protein 2	D1 Chromosomal Protein	Satellite DNA-binding
Sop2	Actin related protein 2/3	CycK	Cyclin K
Cct5	T-complex Chaperonin 5	Hyd (Hyperplastic Disc)	E3-Ub-Ligase
Int6	Proto-oncogene	Scf/DCB-45 (CK2-α)	Chromatin Organization
Tcp1-ζ	HSP60-family	CG6724	WD40 repeats similar to Gβ
Arc-p34	Neuronal development	XNP	Neuronal development
Smg5	Nonsense mediated decay		
**Interacting Partners Unique to Dm-CK2-β′**			
**Protein**	**Function**	**Protein**	**Function**
Porin	Mitochondrial OM channel	awd/abnormal wing discs	Nucleotide Kinase
Chd64	Juvenile Hormone Signaling	EB1	Myosin Binding
Fimbrin	Female meiosis	Smt3/SUMO	SUMO family
FK506-bp2	DNA Damage Response	Nlp/CRP1	Nucleoplasmin
Annexin B10	Annexin Family	PCNA/Mus209	DNA-Replication
**Interacting Partners Common to Dm-CK2-β and Dm-CK2-β′**			
**Protein**	**Function**	**Protein**	**Function**
Fax	Axon connectivity	Nopp140	Cajal body protein
EloB/Elongin-B	Wing cell identity	Otefin	Germline stem cell renewal

Interactions shared with CK2-α in the DPiM database or those revealed by genetic studies are highlighted in red. Unnamed genes are indicated by their annotation symbol (CG#).

**Table 5:** Genes expressed in the developing eye with conserved CK2 sites.

Gene	WebLogo of CK2 Site(s)	Function
Acinus (Acn)	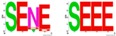	RNA splicing
Anterior Open (Aop)	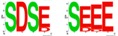	Transcription Factor
Asteroid (Ast)	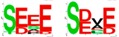	EGFR signaling
AXIN1 upregulated 1 (Axud1)	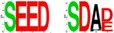	Cell proliferation
Cadherin 86C (Cad86C)	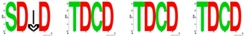	Cell adhesion/signaling
Cadherin N (CadN)	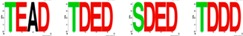	Cell adhesion/signaling
Capicua (Cic)	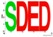	HMG family Transcription Factor
Claspin	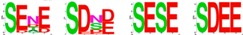	ATR-Chk1 checkpoint pathway
Cubitus Interruptus (Ci)	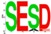	Transcription Factor
Cullin 1 (Cul1)	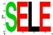	Ubiquitin Ligase
Cullin 3 (Cul3)	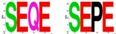	Ubiquitin Ligase
Decapo (Dap)	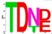	CDK inhibitor
Daughter of Sevenless (Dos)	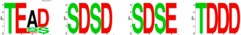	Sevenless RTK signaling
Decay	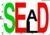	Regulator of apoptosis
Distal Antenna (Dan)	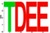	Transcription Factor
Distal antenna related (Danr)	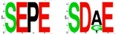	Transcription Factor
Domino (Dom)	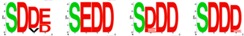	SNF2/RAD54 helicase family
Ebi	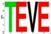	Chromatin binding
EGF-Receptor (EGFR)	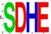	RTK signaling
ELAV	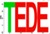	Neurogenesis
Eyegone (Eyg)	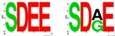	Pax family transcription factor
Eyeless (Ey)	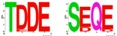	Transcription factor
Eyes Absent (Eya)	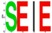	Transcription factor
Fat Facets (Faf)	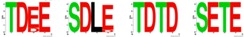	Ubiquitin Ligase
Garnet (G)	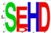	Clathrin coatomer adaptor
Glass (Gl)	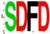	Transcription factor
Golden Goal (Gogo)	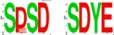	Axon guidance
GP150	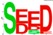	Eye development
Head involution defective (Hid)	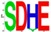	Cell death
Homeodomain interacting Kinase (HipK)	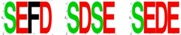	Eye development
IP3-Receptor	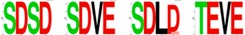	Inositol 1,4,5-tris-phosphate Receptor
Kismet (Kis)	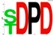	Transcription factor
Klarsicht (Klar)	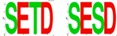	Kinesin binding
Klumpfuss (Klu)	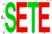	Zinc finger protein
Liprin-γ	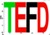	Sterile α motif
Liquid Facets (Lqf)	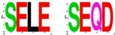	Ubiquitin binding and eye development
Neuralized (Neur)	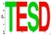	E3 ubiquitin ligase
Osa	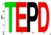	Transcription coactivator
PDGF/VEGF related factor 1	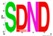	Cell signaling
Pointed (Pnt)	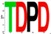	Transcription factor
Prickle (Pk)	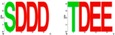	Regulates planar cell polarity
RapGAP1	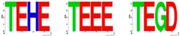	GTPase activating protein
Regulator of eph expression (Reph)	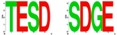	Ephrin signaling
Ret Oncogene (Ret)	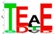	RTK signaling
Scribbler (Sbb)	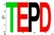	Transcription factor
Serrate (Ser)	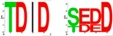	Notch signaling
Seven in absentia (Sina)	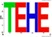	Regulation of R7 differentiation
Shaven (dPax2)	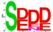	D-Pax2 family transcription factor
Snf5-related 1 (Snr1)	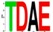	Chromatin structure
Sine Oculis (SO)	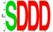	Transcription factor
SoxNeuro (SoxN)	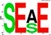	Transcription factor
Spineless	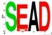	Regulates Rhodopsin expression
Spinster	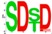	Regulates TGF-β/BMP signaling
Star	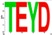	EGF signaling and eye development
Sugarless	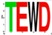	Signaling in eye development
Target of wit (Twit)	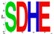	Eye development
Terribly reduced optic lobe (Trol)	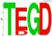	Cell polarity and signaling
Tolkin (Tok)	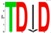	Negative regulator of gene expression
α-catenin	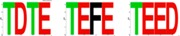	Actin binding
